# Burnout, Depression, and Anxiety Levels among Healthcare Workers Serving Children with Autism Spectrum Disorder

**DOI:** 10.3390/bs12010015

**Published:** 2022-01-15

**Authors:** Shuliweeh Alenezi, Ahmad Almadani, Maram Al Tuwariqi, Fahad Alzahrani, Meshari Alshabri, Mohammed Khoja, Khalid Al Dakheel, Khalil Alghalayini, Norah Alkadi, Shahad Aljebreen, Razan Alzahrani

**Affiliations:** 1 Department of Psychiatry, College of Medicine, King Saud University, Riyadh 11451, Saudi Arabia; ahalmadani@ksu.edu.sa (A.A.); Maltuwairqi@ksu.edu.sa (M.A.T.); Fahad.K.Alzahrani@gmail.com (F.A.); Meshari1418@gmail.com (M.A.); mtkoja2@gmail.com (M.K.); khalid.a.dakheel@gmail.com (K.A.D.); Khaleel.Ghalayini@gmail.com (K.A.); norah.alkadi@gmail.com (N.A.); shahadaljabreen@gmail.com (S.A.); Razanalz127@gmail.com (R.A.); 2 Department of Psychiatry, King Saud University Medical City, King Saud University, Riyadh 11362, Saudi Arabia; 3 SABIC Psychological Health Research and Applications Chair (SPHRAC), Department of Psychiatry, College of Medicine, King Saud University, Riyadh 12372, Saudi Arabia

**Keywords:** burnout, healthcare, depression, anxiety, autism spectrum disorder

## Abstract

Burnout in healthcare workers (HCWs) is defined as a state of emotional, physical, and mental exhaustion that results from unmanaged, excessive, and long-term workplace stressors. This study aims to assess the prevalence of burnout and the levels of anxiety and depression among HCWs who primarily work with children who have autism spectrum disorder (ASD). A quantitative cross-sectional survey was conducted utilizing the Arabic version of the Maslach Burnout Inventory (MBI), Areas of Worklife Survey (AWS), Patient Health Questionnaire for Generalized Anxiety Disorder (GAD-7), and Patient Health Questionnaire for Depression (PHQ-9). Among the 381 participants working in autism centers, the majority were young Saudi females (326) working full-time as specialists in the private sector with less than five years of experience. The HCWs’ overall mean scores on the three Maslach Burnout Inventory (MBI) subscales: emotional exhaustion (EE), depersonalization (DP), and personal accomplishment (PA) were 62%, 23.7%, and 76.5%, respectively. A total of 51.4% of HCWs reported moderate to high anxiety levels on GAD-7, and 47.8% showed moderate to very high levels of depression on PHQ-9. The mean perceived EE converged significantly but negatively on their overall mean perceived satisfaction with AWS (*p*-value < 0.001), demonstrating that greater emotional fatigue predicts less satisfaction with their work. The PA scores correlated significantly and positively with their overall mean satisfaction with their AWS score (*p*-value < 0.001). Considering sociodemographic variables, HCWs aged between 20–29 years have significantly lower mean PA scores than HCWs aged thirty and older (*p* = 0.007). Also, male HCWs perceived significantly higher work-related DP than females. More research is required to determine the nature of variables that contribute to burnout, depression, and anxiety in HCWs helping children with ASD.

## 1. Introduction

The concept of professional burnout was first introduced in the 1960s among healthcare workers at a New York City volunteer clinic [1]. Freudenberger, who was the first to describe professional burnout, defined it as a “multifaceted concept of physical and emotional exhaustion produced by excessive demands on the energy, strength, and resources” [1]. However, 50 years since its introduction, a definition for burnout has yet to be agreed upon. Lisa S. Rotenstein et al., 2018, found up to 142 different definitions used in the literature [2]. The criteria used to consider whether someone was burnt out were vastly different. As a result, the prevalence of burnout has been estimated to be zero in some studies but eighty percent in others [2]. As no accepted standard exists to measure burnout, several measurements have been created and used in the literature. Among these measurements, the Maslach Burnout Inventory (MBI) has gained the most attention. The MBI contains three major domains: emotional exhaustion (EE), depersonalization (DP), and personal accomplishment (PA) [3]. In the healthcare field, EE and DP are the most common domains used to measure burnout [4]. Several studies have looked into the consequences of burnout. For instance, studies showed that burnout is related to both poor well-being and poor job performance. Moreover, serious mental health concerns, including suicide ideation, depression, and substance use, have been linked to burnout [5,6,7].

Career-wise, burnout has been related to serious thoughts of resignation or dropping out, a decline in professional work effort, unprofessional conduct, lower patient satisfaction, and reduced quality of care [8,9,10]. Regarding the provision of care for different types of disorders, studies have examined which subsets could be at the highest risk of burnout. Studies found that providers serving those with psychological and behavioral disturbances, including individuals with autism spectrum disorder (ASD), have the highest rates of burnout, stress, and resignation compared with providers serving those with other intellectual and physical disabilities [11,12,13,14].

One of the high-risk groups for various mental health issues globally is healthcare workers (HCWs). Papers published on this topic have demonstrated that HCWs are prone to problematic levels of psychological distress [15], anxiety [16], burnout, and emotional exhaustion [17]. Depression, among others (anxiety, burnout, and emotional exhaustion), is the most prevalent. Studies showed that, in high-income countries, the prevalence of depression in HCWs ranged from 21.53% to 32.77% and is significantly higher than that of the general population (4.40% in 2015) [18,19,20,21,22].

Moreover, researchers have disputed whether burnout and depression are similar or different in structure in recent years, as they seemingly share some common features (e.g., loss of interest and impaired concentration). However, the researchers found that the results reached were yet inconclusive, having a difference of opinion regarding the extent to which such an overlap should be expected [23].

From a clinical perspective, burnout and depression tend to overlap with each other both at the etiological level, with unresolvable stress as the nodal (i.e., contributory) factor [24,25], and at the symptom level [26,27]. Contrarily, other researchers believe that burnout and depression are distinct. For instance, Ahola and Hakanen (2007) and Shaufeli and Enzmann (1998) illustrated in their studies that numerous investigators believe that burnout and depression are two distinct constructs and that emotional exhaustion and depression are not related. Some unanswered questions include, to what extent is burnout differentiated from depression, and do they complement each other? Burnout being mistakenly labeled as depression and/or anxiety disorders leads to inappropriate management plans, dictating how researchers must answer these critical questions [23]. If burnout is eventually found to be indistinguishable from depression, then the clinical research on management for depression provides hope for helping working people suffering from burnout [28].

Across several studies on burnout and psychiatry, staff who work in mental health services were found to experience high levels of burnout. Burnout has also been empirically associated with anxiety and other negative conditions [29]. Although the link between anxiety and burnout is not fully understood, the emotional exhaustion domain of burnout correlates closely with the existence of anxiety [23]. For instance, a study conducted in Saudi Arabia showcased that ASD is associated with higher incidences of depression and anxiety among parents and caregivers dealing with those affected by ASD, which ultimately leads to more strain and stress on those caregivers [30]. In other studies, staff in direct contact with patients affected by ASD were found to have higher levels of anxiety and tended to encounter a variety of stressful factors that may lead them to harbor feelings of insecurity and helplessness, due to their inability to control difficult aspects encompassed by caring for these people, thus leaving them feeling emotionally depleted [31]. Moreover, previous studies have reported that caregivers of children with ASD are at a higher risk of developing psychological stress, including anxiety and depression; of having decreased family cohesion; and of having increased somatic complaints compared with caregivers of children with other developmental disabilities. The challenges faced by these caregivers, such as the child’s difficulty with self-care, inability to communicate well, and unpredictable behaviors; social isolation; and a lack of community understanding, may pose as additional stressors, which could potentially lead to higher levels of anxiety and other negative psychological outcomes [32,33].

It has been established that not all patients affected by ASD are equal in terms of the severity of their symptoms. Therefore, caregivers might have to deal with patients who are more difficult. Some individuals with ASD might exhibit more self-destructive and violent behaviors, which is mainly attributed to their degree of communication and flexibility. In addition, caregivers may experience variation in the degree of challenges in facilitating routine daily activities and in seeking eye contact. All of these factors could cause more stress on people providing care to children with ASD [31]. Nevertheless, other factors, such as the high expectations of parents, conflicts, indecisiveness, and work overload, may also put caregivers, specifically the parents of children with ASD, under chronic exposure to stress, which has been observed to affect caregivers in several domains of their lives, such as social isolation and poor mental health [33,34].

Autism was once considered a set of neurodevelopmental disorders known as pervasive developmental disorders (PDD). Three core deficits define these disorders: “impaired communication, impaired reciprocal social interaction, and restricted, repetitive and stereotyped patterns of behaviors or interests” [35]. However, autism was revised as a diagnosis in the latest DSM revisions (DSM-V) and is known as autism spectrum disorder (ASD). Hence, ASD is now seen as a spectrum rather than as an affected disease [36]. To address this complex neurodevelopmental disorder, specialized autism centers have been established in Saudi Arabia to support children with ASD who need personalized intensive programs that include behavioral, educational, and psychological interventions [37].

A systematic review identified the predictors related to employment retention, turnover, burnout, and career satisfaction across samples of behavior technicians working with individuals with ASD. Several factors at the employee as well as organizational levels were classified as predictors of burnout, career satisfaction, and intention to turnover in behavior technicians and showed an increased negative implicit attitude towards patients with ASD and higher levels of burnout. In this study, a frequent turnover of behavior professionals working with those affected by ASD was also showcased to potentially negatively impact organizations, staff, and patients [38]. Another study conducted in northern Saudi Arabia among young adults showcased the prevalence of high EE in 64.1%, high DP in 57.6%, and low PA in 32.8% [39]. Healthcare workers, families, and teachers serving patients with autism spectrum disorder are also thought to suffer from increased rates of burnout compared with the general public [40,41,42].

Given the limited number of researchers examining burnout and its relation to depression and anxiety in Saudi Arabia, the aim of our research is to find the prevalence of burnout and the levels of anxiety and depression among healthcare providers who primarily work with patients suffering from ASD and to assess the scope of its effects. The results of this research will ultimately help raise awareness of this issue and help resolve it in order to provide reliable care for patients and to maintain the mental well-being of healthcare providers.

## 2. Material and Methods

### 2.1. Study Design and Participants

Approval for this quantitative, cross-sectional study was obtained from the Institutional Review Board of the College of Medicine at King Saud University. After obtaining copyrights to use the scales, we incorporated them into an online survey using Google Forms. Then, this link was distributed to 110 autism centers identified via the National Center for Mental Health Promotion and Ministry of Human Resource and Social Development registries in Saudi Arabia. These centers then dispersed the surveys to healthcare professionals who currently work with patients and families suffering from ASD.

### 2.2. Survey Instruments

We used the Maslach Burnout Inventory (MBI), Areas of Worklife Survey (AWS), Patient Health Questionnaire for Generalized Anxiety Disorder (GAD-7), and Patient Health Questionnaire for Depression (PHQ-9). All scales used were originally in Arabic and have been used in numerous studies [3,11,14,30]. Our sampling technique was based on convenience sampling, and, regarding our study participants, the sample included all healthcare workers working with individuals affected by ASD. Participant anonymity was maintained by assigning each participant a code number for the purpose of analysis only.

### 2.3. Statistical Data Analysis

Means and standard deviations were used to describe the continuously measured variables, and frequencies and percentages were used to describe the categorically measured variables. Histograms and the statistical Kolmogorov–Smirnov test of normality were used to assess the statistical normality of the measured outcomes. Cronbach’s alpha test was applied to assess the reliability of the measured questionnaires, and the Closeness-to-Unidimensionality test was applied to the measured questionnaires to assess their unidimensionality. A multiple response dichotomies analysis was applied for variables that could be answered with more than an option. The overall concepts and domains for the Maslach Burnout Inventory (MBI), Areas of Worklife Survey (AWS), Patient Health Questionnaire for Generalized Anxiety Disorder (GAD-7), and Patient Health Questionnaire for Depression (PHQ-9) were measured according to the author’s manual, and the resulting scores were analyzed further with descriptive and bivariate analyses. A bivariate Pearson’s correlation test was used to assess the associations between measured concepts and variables. The multivariable Linear Regression Analysis with least squares estimation was used to regress the mean perceived factors influencing work-related burnout for people overall against their sociodemographic characteristics, work-related factors, and measured depression and anxiety scores, in order to assess what factors may explain why people perceived more or less work-related burnout (namely, emotional exhaustion, depersonalization, accomplishment, and work–life satisfaction). The association between predictor variables and the variation in people’s scores for perceived negative acts was expressed as an unstandardized Beta (β) coefficient with its associated 95% confidence interval. The SPSS IBM V21 commercially available statistical data analysis program [43] and the FACTOR standalone factor analysis program (Release 10.9.01) [44] were used for the statistical analysis of the data, and alpha was considered significant at the 0.050 level.

## 3. Results

### 3.1. Reliability and Factorial Validity Analysis of the Measured Scales

The Cronbach’s alpha test of reliability suggested that the Maslach Burnout Inventory was measured reliably, with Cronbach’s alpha = 0.71, and the Areas of Worklife Survey AWS was also measured reliably, with Cronbach’s alpha = 0.75; additionally, the GAD-7 and PHQ-9 measures of anxiety and depression were measured reliably, with Cronbach’s alpha = 0.93 and 0.91, respectively (see Appendix A Table A1). The statistical normality test findings showed that all the measured concepts were either normally distributed or have approximated normality with minor departures from normality, such as the PHQ and GAD7 total scores.

### 3.2. Healthcare Worker Characteristics

Three-hundred-and-eighty-one health professionals working in various autism centers in Saudi Arabia electively enrolled themselves into the study and completed and returned the online survey. Table 1 shows the resulting findings from the analysis of their sociodemographic characteristics and work-related conditions.

### 3.3. Burnout Indicators

Table 2 displays the HCWs’ overall perceptions of work burnout, work–life balance satisfaction, anxiety, and depression. However, Table A2 (see Appendix A) shows the detailed descriptive scores of the HCWs’ perceptions of burnout for three major indicators.

The HCWs’ overall mean perceived emotional exhaustion (EE) was measured as 3.272 out of 6 points, indicating a substantial overall sense of emotional fatigue experienced by those workers. Additionally, the HCWs’ overall mean perceived sense of work-related depersonalization (DP) was measured as 1.42 points out of 6 points. This result highlights an overall low, albeit not negligible, level of depersonalization perceived by the workers serving children with autism spectrum disorders in general. Interestingly, the HCWs’ overall perceived mean personal accomplishment (PA) was relatively high, with the mean PA score for workers equal to 4.60/6 points, unveiling a substantive sense of personal accomplishment according to HCWs dealing with children diagnosed with ASD in Saudi Arabia.

### 3.4. Perceived Workload

Table A3 (see Appendix A) shows a descriptive analysis of the HCWs’ perceptions on the Areas of Worklife Survey (AWS) indicators.

The HCWs’ overall mean perceived satisfaction with areas of their work and life was measured as 3.32/5 points, highlighting substantive satisfaction with their work–life balance in general, but the area of work–life rated as the most satisfying according to those HCWs was their sense of work-related reward, which received a 3.70/5 mean satisfaction rating; followed by work-related group work and community aspects, which received a 3.63/5 mean satisfaction rating; and work-related control, which received an overall mean satisfaction rating of 3.42/5.

### 3.5. Depression and Anxiety Levels

The HCWs’ overall perceived Generalized Anxiety Disorder (GAD-7) level was rated as 9.99 points out of 

21 points, SD = 6.3 points, highlighting moderate-to-high anxiety among the health personnel working with ASD, in general; however, by considering the levels of the anxiety scores based on the cut-off values for the GAD-7 scale experienced by those HCWs, 24.7% of the workers had no to low anxiety, another 23.9% were considered to have mild anxiety, another 22.8% were considered to have moderate anxiety, and 28.6% had high anxiety levels.

### 3.6. Variables Contributing to Burnout, Depression, and Anxiety

Table A4 (see Appendix A) displays a descriptive analysis of the HCWs’ mean scores on the Generalized Anxiety Disorder-7 (GAD-7) and Patient Health Questionnaire for Depression (PHQ-9) indicators.

The HCWs’ overall mean ADL difficulties with anxiety were rated as 1.23/3 points, in general. Additionally, the HCWs’ overall mean perceived depression (PHQ-9) score was measured as 9.99/27 points, SD = 7.10 points, indicating low perceived anxiety-associated ADL difficulties in general among the autism care centers where the HCWs are residing and working in Saudi Arabia. The HCWs’ overall mean depression associated with ADL difficulty was measured at 1.02/3 points, suggestive of a low level of depression-associated ADL difficulties as well among those healthcare workers in general.

The bivariate Pearson’s correlation test results were summarized in Table A5 (see Appendix A). The Multivariate Linear Regression Analysis was applied to better understand what may explain why people perceived greater or lower perceptions of work-related emotional exhaustion (EE). The results from the multivariate findings (Table 3) show that the HCWs’ sex and age did not converge significantly on their mean perceived EE at the workplace, but that the HCWs’ nationality correlated significantly with their EE perceptions. Non-Saudi employees perceived significantly lower emotional exhaustion compared with Saudi HCWs, on average (beta coefficient = −0.304, *p*-value = 0). Additionally, the HCWs’ mean perceived satisfaction with their workload at their ASD care centers correlated significantly but negatively with their mean perceived EE; as the HCWs’ mean perceived satisfaction with workload increased by one point on the Likert scale, their mean perceived EE decreased by 0.448 points, on average (*p* < 0.001), accounting for the other predictor variables in the analysis. Additionally, the HCWs’ mean perceived satisfaction with work-related fairness converged significantly but negatively on their EE perception score (beta = −0.207, *p* < 0.001).

**Figure 1:**
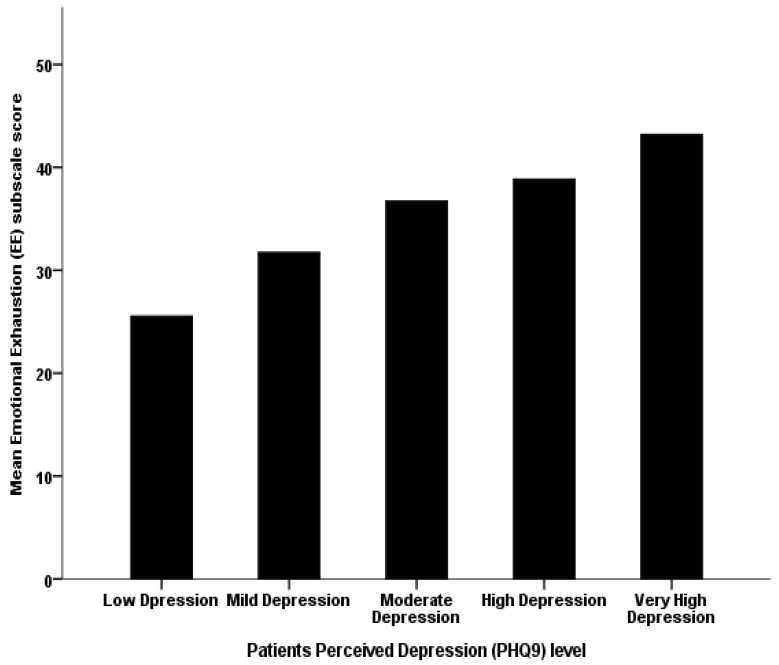
The association between the workers’ categorized perceived depression level and their mean perceived work-related emotional exhaustion.

In addition, the HCWs’ mean perceived PHQ-9 depression scores converged significantly but positively on their mean perceived work-related emotional exhaustion (beta coefficient = 0.058, *p*-value < 0.001); as the HCWs’ perceived depression score increased by one point on the depression PHQ9 total scale score, their corresponding mean perceived work-related emotional fatigue score tended to increase by an increment equal to 0.058, on average (see Figure 1).

**Figure 2:**
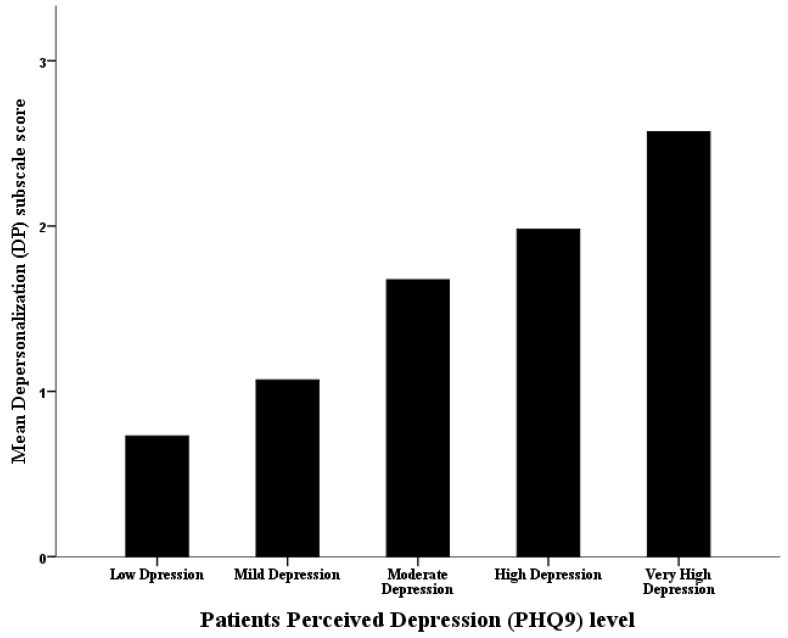
The association between the workers’ categorized perceived depression level and their mean perceived work-related emotional depersonalization.

The analysis of the employees’ mean perceived work-related depersonalization (DP) with Multivariate Linear Regression Analysis (Table 4) showed that male HCWs perceived significantly higher work-related DP compared with females, on average (beta coefficient = 0.639, *p*-value ≤ 0.001), but the HCWs’ age did not converge significantly on their mean perceived DP at the workplace. Additionally, the HCWs’ nationality did not correlate significantly with their work-related DP perceptions either (*p* = 0). However, the HCWs’ mean perceived PHQ-9 depression score converged significantly and positively on their mean perceived work-related sense of depersonalization (beta coefficient = 0.080, *p*-value ≤ 0.001). As the HCWs’ perceived depression scores increased by one point, on average, their corresponding mean perceived work-related depersonalization (DP) scores tended to increase by an increment equal to 0.08 points, on average, too (see Figure 2).

**Figure 3:**
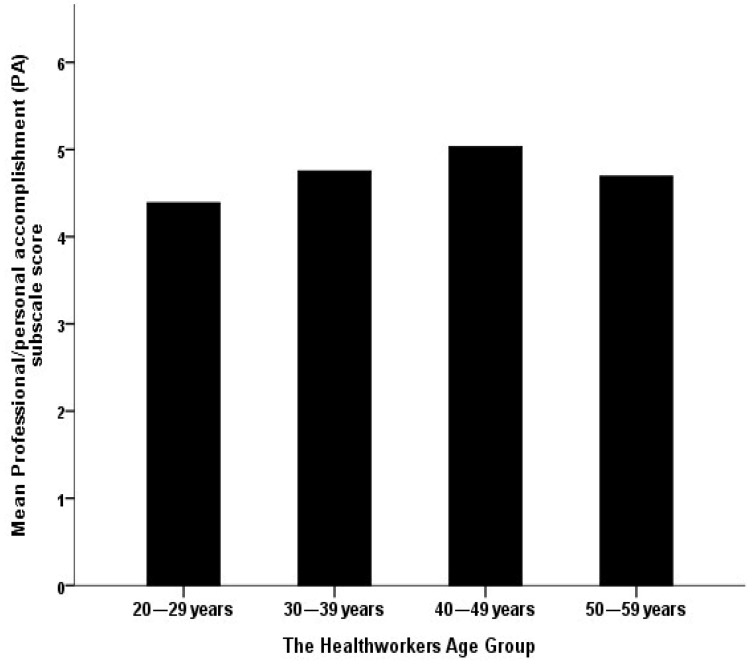
The association between the workers’ age group and their mean perceived work-related personal accomplishment.

The resulting multivariate analysis for the mean perceived personal accomplishment (PA) of HCWs working at ASD care centers, shown in Table 5, suggests that the HCWs’ sex does not correlate significantly with their mean perceived work-related personal accomplishment (PA) score (*p* = 0.914), but workers aged between 20–29 years were found to have significantly lower mean PA scores compared with people aged thirty and older, on average (beta coefficient = −0.238, *p* = 0.007; Figure 3).

Additionally, the HCWs’ total mean perceived depression (PHQ-9) score correlated significantly but negatively with their mean perceived personal accomplishment (beta coefficient = −0.034, *p* < 0.001), accounting for the other predictors in the analysis. The HCWs’ mean perceived satisfaction with work control correlated significantly and positively with their mean perceived work-related personal accomplishment (beta coefficient = 0.264, *p* < 0.001). The HCWs’ mean overall perceived satisfaction with work–life areas was analyzed using multivariate regression (Table 6).

In terms of the HCWs’ mean perceived GAD-7 score, it correlated significantly but negatively with their mean perceived satisfaction with the overall work–life score (beta coefficient = −0.021, *p*-value < 0.001). The HCWs’ mean perceived emotional exhaustion (EE) converged significantly but negatively on their overall mean perceived satisfaction with work–life areas (beta coefficient = −0.184, *p*-value < 0.001), demonstrating that greater perceived emotional fatigue among HCWs working with children with ASD predicts for less satisfaction with their work and life in general. The resulting findings also showed that the HCWs’ mean perceived personal accomplishment correlated significantly and positively with their overall mean satisfaction with their AWS score (beta coefficient = 0.226, *p*-value < 0.001).

## 4. Discussion

This is the first study to address burnout and how it is related to depression and anxiety among health professionals working with children with neurodevelopmental disabilities in Saudi Arabia at a national level. Interestingly, most of the respondents were young Saudi females with fewer than five years of experience working as frontline staff in the private sector. Although potentially considered a response gender bias, we believe this sample could reflect actual practice and the nature of the new initiatives at the governmental level, to empower the private sector to increase services in response to previous reports suggesting an action to help families who are receiving services in neighboring countries [45].

In our sample, the HCWs’ perceptions tended to report emotional exhaustion (EE), evident by feelings that the work was hard. However, other studies conducted in healthcare workers working with individuals with disabilities provided higher feelings of fatigue in the morning when facing another day on the job and at the end of the work day [45]. This difference might be justified, as our sample consists of younger individuals with higher qualifications. On the other hand, our sample showed that depersonalization and carelessness about patients were the lowest-rated perceptions of depersonalization (DP) at work, similar to the results in R. P. Hastings et al., 2004 [46].

The HCWs felt that they were effective at solving patients’ problems, ranked as the top-rated perception of personal accomplishment (PA), followed by feeling that they have a positive impact on other people’s lives by working with them. R. P. Hastings et al., 2018, showed similar results, ranking them second and third highest, respectively.

In the AWS questionnaire, the subscales scores were lower (2.79–3.7) than the sample of Susan L. Ray (3.18 to 3.78) [47]. This could be attributed to our sample being professionals working with ASD patients compared with their sample of caregivers working in mental health facilities. Our findings mostly showed a good impression regarding how HCWs felt about their work. The top indicators in four out of the six dimensions of AWS were positive, and the overall satisfaction using AWS (3.32 out 5) had a slightly higher score than the scores reported in Sarah S. Brom’s and Summer Bottini’s studies (3.28 and 2.95, respectively) [40,48]. We assume that this could be associated with their working experience and that most of them were specialists always dealing with ASD groups. However, our AWS score (3.32) was lower than the score from Susan L. Ray (3.55) [47]. We found that the AWS score is negatively affected by how long work takes to finish, which might be because the HCWs work with a special population that needs more time to show the results desired. This could be resolved by increasing the number of staff and by dividing the workload among them. Additionally, our sample felt that favoritism is one of determining factors in decisions made at work, resulting in a lower score in AWS. We suggest a monthly report including the decisions made, the reasons for those decisions, and the results. Hopefully, this will ensure transparency and help reduce favoritism by showing that the decisions were made on a rational basis rather than a personal one.

Regarding the HCWs’ perceived anxiety, participants most often reported having feelings of nervousness followed by excessive worry and inability to relax, which was as previously observed in other populations [49]. On the other hand, physical complaints including a racing heart rate, clammy extremities, and troubled breathing as well as irrational ruminating thoughts were the obvious manifestations of anxiety in different studies [49]. The indices of stress in our sample appear to be highly exhibited among HCWs as they come across prolonged periods of intense work. More specifically, regarding factors contributing to elevated levels of stress displayed by caregivers of individuals with intellectual disability and autism, the literature identified significant impacts from restricted resource accessibility, poor work environments, a lack of expertise in managing this population, and the susceptibility of the caregiver [50,51]. Moreover, considerable evidence relates the degree of disability and the presence of challenging behaviors to the level of stress expressed by care-providers managing individuals with autism and other developmental disabilities [51,52,53].

In terms of depression, symptoms of fatigue and disturbed sleep patterns were reported more often compared with appetite issues, loss of interest, psychomotor retardation, decreased self-esteem, and suicidal thoughts. Worth mentioning is the fact that depression is more prevalent in caregivers, and even more so in the mothers, of children with autism than with other developmental disabilities [54]. In general, ill mental health affects around 33% of intellectual disability support workers, namely, anxiety and depression [52]. The literature links the increased adversity rate of working with individuals with developmental disability to the high voluntary termination of work [55]. High turnover rates among HCWs in this field are also believed to have an unfavorable impact on the quality of support and on stakeholders’ resources [56].

The overall perception of work burnout in HCWs was strongly related to personal accomplishment (76.5%), followed by emotional exhaustion (62%) and, to a lesser extent, by depersonalization (23.7%). In contrast with other studies, emotional exhaustion ranked first (38%), followed by personal accomplishment (14%) and depersonalization (9%) [40], mainly because, in our sample, the candidates involved themselves with the patients’ problems. In addition, receiving recognition from others as a reward might correlate with high perceptions of personal accomplishment, which was similarly shown in previous studies [40]. This reflection is in agreement with available studies where the personal accomplishment and emotional exhaustion of HCWs showed levels comparable with those of the controls and considerably less depersonalization [55].

Perceived anxiety among HCWs was higher than depression in our study, with percentages of 47.6% and 37%, respectively, in which the majority of anxiety was predominantly of a higher level, while depression is mainly of a moderate level. In comparison to another study [57], this study showed that caregivers experienced more depression compared to anxiety, although the set-up was different as it specifically targeted parents. We believe that caregivers scored high on depression because of their high rate of expectations of cure and the fact that they spend more time with their children as compared to our sample.

Emotional exhaustion (EE), depersonalization (DP), and personal accomplishment (PA) from the Maslach Burnout Inventory (MBI) and Areas of Work Survey (AWS) are used to describe levels of burnout and how mental health is affected by the workplace. The results showed that EE in ASD center HCWs predicted for greater DP but less PA, on average, and was significantly correlated to the amount of time spent by HCWs with children with ASD and their families. This is expected, as HCWs generally are at higher risk of developing emotional exhaustion when working long hours [17,58]. On the other hand, those who had better satisfaction with work-related control, work-related reward, and community, as well as fairness and work-related values, were predicted to experience less EE.

A greater perceived sense of work-related accomplishment predicted for significantly lower feelings of anxiety and depression in general, while the scores for the Patient Health Questionnaire for Depression (PHQ-9) and Generalized Anxiety (GAD-7) and their associated Self-Rated ADL difficulties (Self-Rated ADL difficulty due to Depression (ADLDIFF1) and Self-Rated ADL difficulty due to Anxiety (ADLDIFF2)) had positive significant correlations with EE and DP and negative significant correlations with PA, with no tangible role of the length of work hours on these measures. Interestingly, DP was found to correlate significantly but negatively with all domains of AWS on the bivariate Pearson’s correlation test, while it did not correlate with the overall work–life satisfaction on the work–life areas multivariate regression. High work–life satisfaction exhibits greater PA, while being a front-line employee, a higher GAD-7, EE, and working in a governmental sector are predictors for low levels of overall work–life satisfaction. Note that, with advancements in job rankings and empowerment in the private sector, these rates could go down, along with work–life balance (WLB) interventions.

The results show a significant correlation between all aspects of the MBI (EE, DP, and PA) and HCWs’ mean perceived PHQ-9 depression scores when compared with the general population of HCWs in another study, with a significant correlation between burnout and the prevalence of depression [59]. The complex relationship between depression and burnout and the similarity in symptoms for both might lead to misdiagnoses of burnout cases as depression. Psychiatrists have often debated whether burnout and depression are separate entities or if burnout is a form of depression [23]. A study trying to answer this question concluded that they are “closely related, but that they are certainly not identical twins [60]”. EE is significantly correlated with workload positively and with fairness negatively, similarly to a past study showcasing workload and fairness as the best predictors of emotional exhaustion [40]. DP is significantly correlated with work value positively and with reward negatively, consistent with previous literature establishing a significant correlation of DP with values and reward [40]. While we found a significantly higher mean level of DP among male HCWs, we did not notice a difference in EE or PA scores. The literature suggests that males more frequently report depersonalization than females and that females may be more predisposed to experience EE than males [61]. Lastly, PA is significantly and positively correlated with reward, community value, and work control, similar to a study conducted previously that showed a significant correlation between all AWS scales (except workload) and PA, with values and reward being the greatest predictors of PA [40].

People working with patients who suffer from ASD experience higher levels of burnout, and, thus, they are more prone to developing depression on top of burnout, as the relationship between burnout and the prevalence of depression is clear. This is due to HCWs experiencing higher levels of EE and DP and lower levels of PA. Factors that may aid in decreasing burnout include decreasing the workload or maintaining a workload that is on par with the worker’s expectations. In addition, establishing the organization’s values and what working at the organization is like, specifically during hiring, could be beneficial.

## 5. Conclusions

This study supports the relationship between burnout and depression and anxiety among healthcare professionals working with children with ASD. Interestingly, the majority of participants had less than five years of experience working in this discipline. Although a positive perception of their occupational role was reflected in this study, healthcare providers working with ASD are thought to be more prone to developing burnout compared with the other HCWs, including mental health professionals working with other populations. This study measured burnout using the Maslach Burnout Inventory and the Areas of Worklife Survey, associating them with rates of anxiety and depression. This study indicates that HCWs at ASD centers have adverse psychological states that manifest as anxiety and depression. By measuring these states using the GAD-7, the overall anxiety rates among HCWs were higher than the rates of depression in our study. However, the MBI accounted for a significant correlation between all domains (EE, PE, and PA) and the PHQ-9 depression score, indicated by greater degrees of EE and PE with a diminished sense of PA. On the other hand, feelings of work achievement seemed to be a protective factor against anxiety and depression.

## 6. Implication

We believe that these results may shed some light on the struggles of caring for this vulnerable population. Exploring issues such as burnout and mental health wellbeing is a step towards solving problems in and ultimately improving occupational satisfaction and quality of care. However, further research is needed to address the difficulties of HCWs in the field of developmental disability in order to draw the attention of governments and organizations to support their needs.

## 7. Limitations and Future Directions

We conducted the first study on measuring burnout in HCWs working with an ASD group in our region. As a result, this study should be considered an initial step towards understanding burnout in this kind of group in Saudi Arabia. The main study limitations are the cross-sectional nature of the data, which limits establishing causal relationships or generalizing results. Since participation was voluntary, individuals who were motivated to complete a study examining staff experiences may have different levels of burnout. Our sample was not a unified sample (physicians, nurses, and managers). As a result, comparisons could not be made across the various types of professionals that serve individuals with ASD or across the types of disabilities served. Burnout may differ across these types of professionals and is worthy of examination in future research. Longitudinal and qualitative studies are needed to confirm our findings and to establish the possible risk factors identified in our findings.

## Figures and Tables

**Table 1:** Descriptive analysis of the HCWs’ sociodemographic and professional characteristics; N = 381.

	Frequency	Percentage
Gender		
Female	326	85.6
Male	55	14.4
Age Group		
20–29 years	203	53.3
30–39 years	123	32.3
40–49 years	42	11
50–59 years	13	3.4
Nationality		
Saudi	340	89.2
Non-Saudi	41	10.8
Profession		
Physician	28	7.3
Specialists and nurses	310	81.4
Managers	43	11.3
Type of employment		
Part-time job	57	15
Full-time job	324	85
Work sector		
Private sector	261	68.5
Governmental sector	120	31.5
Current working experience		
≤11 months	90	23.6
1–2 years	71	18.6
3–5 years	122	32
6–10 years	64	16.8
11–15 years	14	3.7
≥16 years	20	5.2
Nature of work with autistic children		
Front-line staff	253	66.4
Management (first-level)	28	7.3
Management (intermediate)	32	8.4
Management (senior)	14	3.7
Supervisor	54	14.2
Percentage of time spent interacting with children with autism spectrum disorders per shift, mean (SD)		54.77 (24.47)

**Table 2:** Descriptive analysis of the workers’ overall perceptions of work burnout, AWS, anxiety, and depression.

	M	SD	Maximum Possible Score
Emotional exhaustion (EE) subscale score	3.72	1.12	0–6 points
Depersonalization (DP) subscale score	1.42	1.22	0–6 points
Professional/personal accomplishment (PA) subscale score	4.59	0.97	0–6 points
Overall satisfaction with areas of work and life	3.32	0.64	1–5 points
Workload AWS subscale score	2.79	0.87	1–5 points
Control AWS subscale score	3.42	1.04	1–5 points
Reward AWS subscale score	3.7	0.97	1–5 points
Community AWS subscale score	3.63	0.83	1–5 points
Fairness AWS subscale score	2.97	0.81	1–5 points
Values AWS subscale score	3.41	0.82	1–5 points
Generalized Anxiety Disorder (GAD-7) score	9.99	6.28	0–21 points
Self-rated ADL difficulty due to anxiety	1.23	1	0–3 points
Patient Health Questionnaire for Depression (PHQ-9) score	9.99	7.06	0–27 points
Self-rated ADL difficulty due to depression	1.02	1	0–3 points

**Table 3:** Multivariate Linear Regression Analysis of the workers’ perceived work-related emotional exhaustion (EE) score.

	Unstandardized Beta Coefficients	95.0% CI for Beta Coefficient		*p*-Value
		Lower Bound	Upper Bound	
(Constant)	5.002	4.465	5.540	<0.001
Sex = male	−0.042	−0.279	0.195	0.728
Age group	−0.111	−0.251	0.028	0.116
Nationality = non-Saudi	−0.304	−0.573	−0.034	0.027
Perceived satisfaction with workload AWS subscale score	−0.448	−0.559	−0.337	<0.001
Perceived work-related fairness AWS subscale score	−0.207	−0.316	−0.099	<0.001
Perceived Patient Health Questionnaire for Depression (PHQ-9) score	0.058	0.044	0.072	<0.001
Current organization work experience	0.085	0.007	0.162	0.033

DV = mean emotional exhaustion (EE) score. Model R-squared = 0.542, adjusted R-squared = 0.530.

**Table 4:** Multivariate Linear Regression Analysis of the workers’ perceived work-related depersonalization (DP) score.

	Unstandardized Beta Coefficients	95.0% CI for Beta Coefficient		*p*-Value
		Lower Bound	Upper Bound	
(Constant)	1.057	0.346	1.768	0.004
Sex = male	0.639	0.342	0.936	<0.001
Age group	−0.055	−0.203	0.092	0.460
Nationality = non-Saudi	−0.223	−0.560	0.113	0.193
Perceived Patient Health Questionnaire for Depression (PHQ-9) score	0.080	0.063	0.096	<0.001
Perceived satisfaction with work-related reward AWS subscale score	−0.202	−0.332	−0.072	0.002
Perceived satisfaction with community benefit AWS subscale score	−0.090	−0.248	0.067	0.261
Perceived work-related value AWS subscale score	0.173	0.029	0.316	0.019
Responsibility role = frontline staff/direct patient carers	0.082	0.005	0.159	0.037

DV = mean perceived depersonalization (DP) score. Model R-squared = 0.33, adjusted R-squared = 0.32.

**Table 5:** Multivariate Linear Regression Analysis of the workers’ perceived personal accomplishment (PA) score.

	Unstandardized Beta Coefficients	95.0% CI for Beta Coefficient		*p*-Value
		Lower Bound	Upper Bound	
(Constant)	3.309	2.654	3.963	<0.001
Sex = male	−0.013	−0.249	0.223	0.914
Age group ≤ 29 years	−0.238	−0.412	−0.064	0.007
Nationality = non-Saudi	0.150	−0.125	0.424	0.284
Perceived Patient Health Questionnaire for Depression (PHQ-9) score	−0.034	−0.048	−0.019	<0.001
Perceived satisfaction with workload AWS subscale score	−0.104	−0.219	0.011	0.077
Perceived work control AWS subscale score	0.264	0.160	0.368	<0.001
Perceived work-related fairness AWS subscale score	−0.071	−0.198	0.056	0.270
Perceived work-related reward AWS subscale score	0.116	0.007	0.224	0.036
Perceived community value AWS subscale score	0.191	0.062	0.319	0.004
Percentage of time per shift spent interacting with autistic children	0.004	0.000	0.007	0.031

DV = mean perceived personal accomplishment (PA) score. Model R-squared = 0.33, adjusted R-squared = 0.32.

**Table 6:** Multivariate Linear Regression Analysis of the workers’ perceived overall mean work–life satisfaction.

	Unstandardized Beta Coefficients	95.0% CI for Beta Coefficient		*p*-Value
		Lower Bound	Upper Bound	
(Constant)	3.408	3.057	3.758	<0.001
Sex = male	0.017	−0.129	0.163	0.818
Age group	−0.020	−0.093	0.052	0.583
Nature of work = frontline staff	−0.145	−0.262	−0.028	0.016
Perceived Generalized Anxiety Disorder (GAD-7) score	−0.021	−0.031	−0.010	<0.001
Perceived work-related emotional exhaustion (EE) subscale score	−0.184	−0.240	−0.128	<0.001
Perceived work-related depersonalization (DP) subscale score	0.006	−0.042	0.054	0.808
Perceived personal accomplishment (PA) subscale score	0.226	0.170	0.283	<0.001
Work sector = governmental	−0.177	−0.288	−0.066	0.002
Percentage of time per shift spent interacting with children with autism spectrum disorders	−0.001	−0.003	0.001	0.286

DV = mean score for workers’ overall satisfaction with their work and life. Model R-squared = 0.47, adjusted R-squared = 0.46.

## Data Availability

All the data for this study will be made available upon reasonable request.

## Appendix A

**Table A1:** Reliability analysis of the employees’ measured perceptions; N = 381.

	Number of Items	Cronbach’s Alpha
Maslach Burnout Inventory—medical personnel version	22	0.71
Areas of Worklife Survey (AWS)	28	0.75
Generalized Anxiety Disorder (GAD-7)	7	0.93
Patient Health Questionnaire for Depression (PHQ-9)	9	0.91

**Table A2:** Descriptive analysis of the HCWs’ perceptions of work burnout indicators.

	M	SD	Rank
Emotional Exhaustion			
I feel emotionally drained from my work	3.62	1.9	6
2. I feel used up at the end of the workday	4.26	1.72	4
3. I feel fatigued when I wake up in the morning and have to face another day on the job	3.28	2.06	7
4. Working with people all day is really a strain for me	4.28	1.77	3
I feel burnt out from my work	2.91	2.12	8
5. I feel frustrated by my job	1.93	2.06	9
6. I feel that I am working too hard at my job	4.93	1.36	1
7. Working with people directly puts too much stress on me	4.61	1.61	2
8. I feel that I am at the end of my rope	3.7	1.97	5
Depersonalization			
I feel that I treat some patients as if they are impersonal objects	0.7	1.33	4
2. I have become more callous towards people since I took this job	1.59	1.96	2
3. I worry that this job is hardening me emotionally	1.47	2.03	3
4. I do not really care what happens to some patients	0.61	1.27	5
5. I feel that patients blame me for some of their problems	2.71	2.14	1
Personal Accomplishment			
I can easily understand how my patients feel about things	4.61	1.43	5
2. I deal with the problems of my patients very effectively	5.29	1.08	1
3. I feel that I am positively influencing other people’s lives through my work	5.12	1.31	2
4. I feel very energetic	3.69	1.77	8
5. I can easily create a relaxed atmosphere with my patients	4.63	1.52	4
6. I feel exhilarated after working closely with my patients	4.41	1.57	6
7. I have accomplished many worthwhile things in this job	4.68	1.55	3
8. In my work, I deal with emotional problems very calmly	4.26	1.55	7

M = mean score, SD = standard deviation. Rank = ascending mean rank.

**Table A3:** Descriptive analysis of the HCWs’ perceptions on the Areas of Worklife Survey (AWS) indicators.

	M	SD	Rank
Workload			
I do not have time to complete work that must be finished	3.04	1.25	4
2. I work intensely for prolonged periods of time	3.79	1.08	1
3. I have so much work to carry out for the job that it takes away from my personal life	3.5	1.3	2
4. I have enough time to complete important tasks for my job	3.26	1.21	3
5. I leave my work behind when I go home at the end of the workday	3	1.45	5
Control			
I have control over how I conduct my work	3.79	1.07	1
2. I can influence management to obtain the equipment and space that I need for my work	3.03	1.35	4
3. I have professional autonomy (independence) in my work	3.44	1.32	2
4. I have influence in the decisions affecting my work	3.41	1.22	3
Reward			
I receive recognition from others for my work	3.96	1.11	1
2. My work is appreciated	3.72	1.24	2
3. My efforts usually go unnoticed	2.46	1.27	3
4. I am not recognized for all of the things I contribute	2.39	1.26	4
Community			
People trust one another fairly when fulfilling their roles	3.48	1.05	4
2. I am a member of a supportive work group	3.57	1.24	3
3. Members of my work group cooperate with one another	3.82	1.08	1
4. Members of my work group communicate openly	3.63	1.14	2
5. I do not feel close to my colleagues	2.33	1.22	5
Fairness			
Resources are allocated fairly here	2.98	1.19	4
2. Opportunities are decided solely on merit	3.01	1.27	3
3. Effective appeal procedures are available when I question the fairness of a decision	2.7	1.23	6
4. Management treats all employees fairly	3.08	1.37	2
5. Favoritism determines how decisions are made at work	3.13	1.28	1
6. Rather than what you know, who you know determines your career here	2.85	1.33	5
Value			
My values and the organization’s values are aligned	3.51	1.16	1
2. The organization’s goals influence my day-to-day work activities	3.33	1.19	3
3. My personal career goals are consistent with the organization’s stated goals	3.32	1.21	4
4. The organization is committed to quality	3.48	1.25	2

**Table A4:** Descriptive analysis of the HCWs’ scores on the Generalized Anxiety Disorder-7 and Patient Health Questionnaire for Depression (PHQ-9) indicators.

	M	SD	RANK
GAD-7			
Feeling nervous, anxious, or on edge	1.69	1.01	1
2. Not being able to stop or control worrying	1.34	1.06	5
3. Worrying too much about different things	1.64	1.07	2
4. Trouble relaxing	1.6	1.05	3
5. Being so restless, that sitting still is hard	1.23	1.14	6
6. Becoming easily annoyed or irritable	1.41	1.08	4
7. Feeling afraid that something awful might happen	1.09	1.13	7
PHQ-9			
Little interest or pleasure in carryout out tasks	1.27	1.03	4
2. Feeling down, depressed, or hopeless	1.17	1.04	5
3. Trouble falling or staying asleep, or sleeping too much	1.35	1.08	2
4. Feeling tired or having little energy	1.61	1.03	1
5. Poor appetite or over-eating	1.31	1.1	3
6. Feeling bad about yourself—or that you are a failure or have let yourself or your family down	0.91	1.07	7
7. Trouble concentrating on things, such as reading the newspaper or watching television	1.13	1.11	6
8. Moving or speaking so slowly that other people could have noticed, or the opposite—being so fidgety or restless that you have been moving around a lot more than usual	0.82	1.02	8
9. Thoughts that you would be better off dead or of hurting yourself in some way	0.4	0.81	9

**Table A5:** Bivariate Pearson’s correlations between the workers’ overall perceptions.

	Emotional Exhaustion	DP	PA	Workload	Control	Reward	Community	Fairness	Value	GAD-7	PHQ-9	ADLDIFF1	ADLDIFF2
Depersonalization (DP) subscale score	0.446 **												
Professional/personal accomplishment (PA) subscale score	−0.149 **	−0.322 **											
Workload AWS subscale score	−0.583 **	−0.284 **	0.206 **										
Control AWS subscale score	−0.344 **	−0.209 **	0.443 **	0.386 **									
Reward AWS subscale score	−0.303 **	−0.318 **	0.396 **	0.294 **	0.494 **								
Community AWS subscale score	−0.343 **	−0.274 **	0.407 **	0.351 **	0.498 **	0.521 **							
Fairness AWS subscale score	−0.377 **	−0.211 **	0.255 **	0.308 **	0.519 **	0.452 **	0.471 **						
Values AWS subscale score	−0.247 **	−0.073	0.277 **	0.202 **	0.470 **	0.375 **	0.470 **	0.510 **					
Generalized Anxiety Disorder (GAD-7) score	0.583 **	0.399 **	−0.382 **	−0.524 **	−0.392 **	−0.389 **	−0.390 **	−0.328 **	−0.266 **				
Patient Health Questionnaire for Depression (PHQ-9) score	0.593 **	0.509 **	−0.374 **	−0.514 **	−0.293 **	−0.381 **	−0.426 **	−0.322 **	−0.250 **	0.755 **			
Self-Rated ADL Difficulty due to Depression (ADLDIFF1)	0.459 **	0.372 **	−0.287 **	−0.397 **	−0.291 **	−0.275 **	−0.329 **	−0.286 **	−0.197 **	0.630 **	0.772 **		
Self-Rated ADL Difficulty due to Anxiety (ADLDIFF2)	0.470 **	0.372 **	−0.319 **	−0.395 **	−0.327 **	−0.331 **	−0.337 **	−0.331 **	−0.226 **	0.743 **	0.694 **	0.703 **	
Percentage of time per shift spent interacting with children with autism spectrum disorder	0.123 **	0.038	0.089	−0.169 **	−0.022	−0.055	−0.029	−0.092	−0.025	0.087	0.091	0.024	0.104 **

** Correlation is significant at the 0.01 level (two-tailed).

## References

[1] Freudenberger H.J. (1974). Staff Burn-Out. J. Soc. Issues.

[2] Rotenstein L.S., Torre M., Ramos M.A., Rosales R.C., Guille C., Sen S., Mata D.A. (2018). Prevalence of Burnout Among Physicians: A Systematic Review. JAMA.

[3] Maslach C., Jackson S.E. (1981). The measurement of experienced burnout. J. Organ. Behav..

[4] Hewitt D.B., Ellis R.J., Hu Y.-Y., Cheung E.O., Moskowitz J.T., Agarwal G., Bilimoria K.Y. (2020). Evaluating the Association of Multiple Burnout Definitions and Thresholds with Prevalence and Outcomes. JAMA Surg..

[5] Dyrbye L.N., Thomas M.R., Massie F.S., Power D.V., Eacker A., Harper W., Durning S., Moutier C., Szydlo D.W., Novotny P.J. (2008). Burnout and Suicidal Ideation among U.S. Medical Students. Ann. Intern. Med..

[6] Shanafelt T.D., Balch C.M., Dyrbye L., Bechamps G., Russell T., Satele D., Rummans T., Swartz K., Novotny P.J., Sloan J. (2011). Special Report: Suicidal Ideation among American Surgeons. Arch. Surg..

[7] Oreskovich M.R., Kaups K.L., Balch C.M., Hanks J.B., Satele D., Sloan J., Meredith C., Buhl A., Dyrbye L.N., Shanafelt T.D. (2012). Prevalence of Alcohol Use Disorders Among American Surgeons. Arch. Surg..

[8] Halbesleben J.R.B., Rathert C. (2008). Linking physician burnout and patient outcomes: Exploring the Dyadic Relationship between Physicians and Patients. Health Care Manag. Rev..

[9] Tawfik D.S., Scheid A., Profit J., Shanafelt T., Trockel M., Adair K.C., Sexton J.B., Ioannidis J.P. (2019). Evidence Relating Health Care Provider Burnout and Quality of Care: A Systematic Review and Meta-Analysis. Ann. Intern. Med..

[10] Dyrbye L.N., Thomas M.R., Power D.V., Durning S., Moutier C., Massie F.S., Harper W., Eacker A., Szydlo D.W., Sloan J.A. (2010). Burnout and Serious Thoughts of Dropping Out of Medical School: A Multi-Institutional Study. Acad. Med..

[11] Banks S.R., Necco E.G. (1990). The Effects of Special Education Category and Type of Training on Job Burnout in Special Education Teachers. Teach. Educ. Spec. Educ. J. Teach. Educ. Div. Counc. Except. Child..

[12] Nichols A.S., Sosnowsky F.L. (2002). Burnout Among Special Education Teachers in Self-Contained Cross-Categorical Classrooms. Teach. Educ. Spec. Educ. J. Teach. Educ. Div. Counc. Except. Child..

[13] Hastings R.P., Brown T. (2002). Coping Strategies and the Impact of Challenging Behaviors on Special Educators’ Burnout. Ment. Retard..

[14] Zabel R.H., Zabel M.K. (1982). Factors in Burnout among Teachers of Exceptional Children. Except. Child..

[15] Masanotti G.M., Paolucci S., Abbafati E., Serratore C., Caricato M. (2020). Sense of Coherence in Nurses: A Systematic Review. Int. J. Environ. Res. Public Health.

[16] Gómez-Salgado J., Domínguez-Salas S., Romero-Martín M., Ortega-Moreno M., García-Iglesias J.J., Ruiz-Frutos C. (2020). Sense of Coherence and Psychological Distress Among Healthcare Workers During the COVID-19 Pandemic in Spain. Sustainability.

[17] Burton A., Burgess C., Dean S., Koutsopoulou G.Z., Hugh-Jones S. (2017). How Effective are Mindfulness-Based Interventions for Reducing Stress Among Healthcare Professionals? A Systematic Review and Meta-Analysis. Stress Health.

[18] World Health Organization (WHO) (2017). Depression and Other Common Mental Disorders: Global Health Estimates.

[19] Mata D.A., Ramos M.A., Bansal N., Khan R., Guille C., Di Angelantonio E., Sen S. (2015). Prevalence of Depression and Depressive Symptoms Among Resident Physicians a Systematic Review and Meta-Analysis. JAMA J. Am. Med. Assoc..

[20] Maharaj S., Lees T., Lal S. (2019). Prevalence and Risk Factors of Depression, Anxiety, and Stress in a Cohort of Australian Nurses. Int. J. Environ. Res. Public Health.

[21] De Oliveira G.S., Chang R., Fitzgerald P.C., Almeida M.D., Castro-Alves L.S., Ahmad S., McCarthy R.J. (2013). The Prevalence of Burnout and Depression and Their Association with Adherence to Safety and Practice Standards: A Survey of United States Anesthesiology Trainees. Anesth. Analg..

[22] Melnyk B.M., Orsolini L., Tan A., Arslanian-Engoren C., Melkus G.D., Dunbar-Jacob J., Rice V.H., Millan A., Dunbar S.B., Braun L.T. (2018). A National Study Links Nurses’ Physical and Mental Health to Medical Errors and Perceived Worksite Wellness. J. Occup. Environ. Med..

[23] Koutsimani P., Montgomery A., Georganta K. (2019). The Relationship Between Burnout, Depression, and Anxiety: A Systematic Review and Meta-Analysis. Front. Psychol..

[24] Hätinen M., Kinnunen U., Pekkonen M., Kalimo R. (2007). Comparing two burnout interventions: Perceived job control mediates decreases in burnout. Int. J. Stress Manag..

[25] Rydmark I., Wahlberg K., Ghatan P.H., Modell S., Nygren Å., Ingvar M., Åsberg M., Heilig M. (2006). Neuroendocrine, Cognitive and Structural Imaging Characteristics of Women on Longterm Sickleave with Job Stress–Induced Depression. Biol. Psychiatry.

[26] Bianchi R., Boffy C., Hingray C., Truchot D., Laurent E. (2013). Comparative symptomatology of burnout and depression. J. Health Psychol..

[27] Kahn J.P. (2008). Diagnosis and Referral of Workplace Depression. J. Occup. Environ. Med..

[28] Bianchi R., Schonfeld I.S., Laurent E. (2014). Is burnout a depressive disorder? A reexamination with special focus on atypical depression. Int. J. Stress Manag..

[29] Papathanasiou I.V., Tsaras K., Kleisiaris C.F., Fradelos E.C., Tsaloglidou A., Damigos D., Vlamos P. (2017). Anxiety and Depression in Staff of Mental Units: The Role of Burnout. Advances in Experimental Medicine and Biology.

[30] Almansour M.A., Alateeq M.A., Alzahrani M.K., Algeffari M.A., Alhomaidan H.T. (2013). Depression and anxiety among parents and caregivers of autistic spectral disorder children. Neurosciences.

[31] Manzano-García G., Ayala J.-C. (2017). Relationship between Psychological Capital and Psychological Well-Being of Direct Support Staff of Specialist Autism Services. The Mediator Role of Burnout. Front. Psychol..

[32] Adib N.A.N., Ibrahim M.I., Ab Rahman A., Bakar R.S., Yahaya N.A., Hussin S., Mansor W.N.A.W. (2019). Perceived Stress among Caregivers of Children with Autism Spectrum Disorder: A State-Wide Study. Int. J. Environ. Res. Public Health.

[33] Rafaq F., Ijaz S., Latif S., Ijaz S. (2020). The association between psychological capital, mental health and burnout among specialists working in autism centers in Pakistan. J. Environ. Occup. Health.

[34] Lai W.W., Oei T.P.S. (2014). Coping in Parents and Caregivers of Children with Autism Spectrum Disorders (ASD): A Review. Rev. J. Autism Dev. Disord..

[35] Faras H., Al Ateeqi N., Tidmarsh L. (2010). Autism spectrum disorders. Ann. Saudi Med..

[36] Babatin A.M., Alzahrani B.S., Jan F.M., Alkarimi E.H., Jan M.M. (2016). The availability of services for children with autism spectrum disorder in a Saudi population. Neurosciences.

[37] American Psychiatric Association (2013). Diagnostic and Statistical Manual of Mental Disorders.

[38] Novack M.N., Dixon D.R. (2019). Predictors of Burnout, Job Satisfaction, and Turnover in Behavior Technicians Working with Individuals with Autism Spectrum Disorder. Rev. J. Autism Dev. Disord..

[39] Alrayyes S., Dar U.F., Alrayes M., Alghutayghit A., Alrayyes N. (2020). Burnout and imposter syndrome among Saudi young adults: The Strings in the Puppet Show of Psychological Morbidity. Saudi Med. J..

[40] Bottini S., Wiseman K., Gillis J. (2020). Burnout in providers serving individuals with ASD: The impact of the workplace. Res. Dev. Disabil..

[41] Elsabbagh M., Divan G., Koh Y.-J., Kim Y.S., Kauchali S., Marcín C., Montiel-Nava C., Patel V., Paula C.S., Wang C. (2012). Global Prevalence of Autism and Other Pervasive Developmental Disorders. Autism Res..

[42] Salhia H.O., Al-Nasser L.A., Taher L.S., Al-Khathaami A.M., El-Metwally A.A. (2014). Systemic review of the epidemiology of autism in Arab Gulf countries. Neurosciences.

[43] IBM Corporation (2012). Released IBM SPSS Statistics for Windows, Version 21.

[44] FACTOR Standalone Factor Analysis Program (Release 10.9.01). https://psico.fcep.urv.cat/utilitats/factor/Download.html.

[45] Al Nemary F.M., Aldhalaan H.M., Simon-Cereijido G., AlNemary F.M. (2017). Services for children with autism in the Kingdom of Saudi Arabia. Autism.

[46] Hastings R.P., Horne S., Mitchell G. (2004). Burnout in direct care staff in intellectual disability services: A factor analytic study of the Maslach Burnout Inventory. J. Intellect. Disabil. Res..

[47] Ray S.L., Wong C., White D., Heaslip K. (2013). Compassion satisfaction, compassion fatigue, work life conditions, and burnout among frontline mental health care professionals. Traumatology.

[48] Brom S.S., Buruck G., Horváth I., Richter P., Leiter M.P. (2015). Areas of worklife as predictors of occupational health—A validation study in two German samples. Burn. Res..

[49] Al-Farsi O.A., Al-Farsi Y.M., Al-Sharbati M.M., Al-Adawi S. (2016). Stress, anxiety, and depression among parents of children with autism spectrum disorder in Oman: A case-control study. Neuropsychiatr. Dis. Treat..

[50] Eliacin J., Flanagan M., Monroe-DeVita M., Wasmuth S., Salyers M.P., Rollins A.L. (2018). Social capital and burnout among mental healthcare providers. J. Ment. Health.

[51] Singh N.N., Lancioni G.E., Medvedev O.N., Hwang Y.-S., Myers R.E., Townshend K. (2020). Using mindfulness to improve quality of life in caregivers of individuals with intellectual disabilities and autism spectrum disorder. Int. J. Dev. Disabil..

[52] Mutkins E., Brown R.F., Thorsteinsson E.B. (2011). Stress, depression, workplace and social supports and burnout in intellectual disability support staff. J. Intellect. Disabil. Res..

[53] Lecavalier L., Leone S., Wiltz J. (2006). The impact of behaviour problems on caregiver stress in young people with autism spectrum disorders. J. Intellect. Disabil. Res..

[54] Baykal S., Karakurt M.N., Çakır M., Karabekiroğlu K. (2019). An Examination of the Relations Between Symptom Distributions in Children Diagnosed with Autism and Caregiver Burden, Anxiety and Depression Levels. Community Ment. Health J..

[55] Nevill R.E.A., Havercamp S. (2018). Retention, Resilience, and Burnout of Staff Caregivers for Aggressive Adults with DD. Abstracts International: Section B: The Sciences and Engineering. Ph.D. Thesis.

[56] McKillop J.M., Minnes P. (2011). Occupational Satisfaction, Strain, and Intention to Quit among Direct Care Providers Assisting Individuals with Developmental Disabilities. J. Dev. Disabil..

[57] Scherer N., Verhey I., Kuper H. (2019). Depression and anxiety in parents of children with intellectual and developmental disabilities: A systematic review and meta-analysis. PLoS ONE.

[58] Li Z., Dai J., Wu N., Jia Y., Gao J., Fu H. (2019). Effect of Long Working Hours on Depression and Mental Well-Being among Employees in Shanghai: The Role of Having Leisure Hobbies. Int. J. Environ. Res. Public Health.

[59] Lacy B.E., Chan J.L. (2018). Physician Burnout: The Hidden Health Care Crisis. Clin. Gastroenterol. Hepatol..

[60] Brenninkmeyer V., Van Yperen N.W., Buunk B.P. (2001). Burnout and depression are not identical twins: Is decline of superiority a distinguishing feature?. Personal. Individ. Differ..

[61] Adam S., Mohos A., Kalabay L., Torzsa P. (2018). Potential correlates of burnout among general practitioners and residents in Hungary: The significant role of gender, age, dependant care and experience. BMC Fam. Pract..

